# Phosphorylation of Kindlins and the Control of Integrin Function

**DOI:** 10.3390/cells10040825

**Published:** 2021-04-07

**Authors:** Katarzyna Bialkowska, Jun Qin, Edward F. Plow

**Affiliations:** Department of Cardiovascular and Metabolic Sciences, Lerner Research Institute, Cleveland Clinic, Cleveland, OH 44195, USA; bialkok@ccf.org (K.B.); qinj@ccf.org (J.Q.)

**Keywords:** kindlins, phosphorylation, post-translational modifications

## Abstract

Integrins serve as conduits for the transmission of information between cells and their extracellular environment. Signaling across integrins is bidirectional, transducing both inside-out and outside-signaling. Integrin activation, a transition from a low affinity/avidity state to a high affinity/avidity state for cognate ligands, is an outcome of inside-signaling. Such activation is particularly important for the recognition of soluble ligands by blood cells but also influences cell-cell and cell-matrix interactions. Integrin activation depends on a complex series of interactions, which both accelerate and inhibit their interconversion from the low to the high affinity/avidity state. There are three components regarded as being most proximately involved in integrin activation: the integrin cytoplasmic tails, talins and kindlins. The participation of each of these molecules in integrin activation is highly regulated by post-translation modifications. The importance of targeted phosphorylation of integrin cytoplasmic tails and talins in integrin activation is well-established, but much less is known about the role of post-translational modification of kindlins. The kindlins, a three-member family of 4.1-ezrin-radixin-moesin (FERM)-domain proteins in mammals, bind directly to the cytoplasmic tails of integrin beta subunits. This commentary provides a synopsis of the emerging evidence for the role of kindlin phosphorylation in integrin regulation.

## 1. Introduction

Integrins, the subject of this collection, serve as primary portals of communication between cells and their extracellular environment. Information transfer across integrins is bidirectional; signaling is both inside-out and outside-in. Productive inside-out signaling results in integrin activation, a transition from a low affinity/avidity state to a high affinity/avidity state for cognate ligands. Such activation is particularly important for the recognition of soluble ligands by blood cell integrins and for certain cell–cell and cell–matrix interactions (e.g., [1,2,3,4,5]). While many different molecular interactions are engaged as a consequence of integrin activation leading to the formation of nascent focal adhesions and maturation into multi-molecular adhesomes [6], three molecules are now recognized as being key to and directly engaged in the activation of most integrins: (i) the integrin itself where changes are initiated in their cytoplasmic tails (CT) [7]; (ii) talin, a large cytoskeletal protein composed of a 4.1-ezrin-radixin-moesin (FERM) domain that resides in the talin head (talin-H) and a long, multi-helical bundle (the rod domain); and (iii) a kindlin, a three-member family of proteins composed of a FERM domain intersected by a PH domain [8]. Accelerants of integrin activation, such as paxillin [9,10], ADAP [11] and migfillin ([12], and inhibitors of integrin activation, such as ICAP-1 [13], filamin [14,15] and sharpin [16,17], allow for fine-tuning of the integrin activation process.

Bidirectional signaling across integrins relies heavily upon post-translational modifications (PTMs) with protein phosphorylation being particularly prominent. Activation of serine, threonine and tyrosine phosphokinases are associated with ligation of integrins as is the activation of phosphatase dependent pathways. Within the integrin activation triumvirate of integrin β CT, talin and kindlin, each component is subject phosphorylation. Both the α and β CT are susceptible to phosphorylation (e.g., [18,19]). These PTM occur within specific motifs in the beta CT and are involved in the initiation, propagation and termination of outside-in and inside-out signaling. The role of integrin β CT phosphorylation has been considered by excellent reviews [20,21,22]. Similarly, PTM of talin is extensive and was recognized early in the discovery of talin and its role in integrin activation [23,24]. The functional significance of PTM of kindlins controlling integrin signaling is now beginning to be recognized. After a brief summary of the kindlins structure, this commentary focuses on this emerging evidence that PTM of kindlins plays significant roles in integrin activation.

### 1.1. Kindlin Structure and Its Role in Integrin Activation

**Figure 1:**
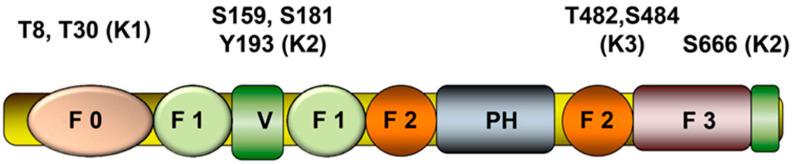
Domain structure of a prototypic kindlin with functionally important phosphorylation sites identified.

The three kindlins (kindlin-1 (K1), (FERMT1), kindlin-2 (K2, FERMT2) and kindlin-3 (K3, FERMT3), found in mammals are products of separate genes [25,26]. They are approximately 50% identical at the amino acid level and are composed of regions of high homology which are interspersed with short hypervariable regions [27]. As FERM domain proteins, each kindlin is composed of the typical F1, F2 and F3 subdomains, which is preceded by an F0 subdomain and ends in a short sequence of ~8 amino acids which is important for integrin activation by K2 [28] (see Figure 1). Distinguishing kindlins from other FERM domain proteins is the insertion of a PH domain into the F2 subdomain. The primary integrin-binding site in kindlins resides in the F3 (PTB-like) subdomain. The integrin-binding function of all three kindlins can be blunted by mutation of a QW sequence, K1QW^612^, K2QW^615^ and K3QW^598^ to alanines [29,30]. The lipid-binding properties of the PH domain and a second lipid-binding site in F0 [31,32] help to target K2 to membranes [33,34], interactions that may influence its integrin co-activator function [35,36,37]. PH domains of all three kindlins are functional lipid binding sites. The FERM domain of kindlins is most homologous to that of talin, and both bind through their F3 subdomains to the short CT of integrin beta subunits. Nevertheless, the binding sites for kindlins and talins in the integrin β CT bring the molecules in close proximity but appear to be non-overlapping [38].

Kindlins are adapter proteins with many different binding partners giving rise to many different cellular responses [39,40]. The descriptions of such binding partners continue to expand but are not the topic of this review. It was originally thought that each kindlin was expressed and exerted its major effects in a tissue-specific manner [41,42,43]: K1 in epithelial cells, K3 in hematopoietic cells, and K2 in most other tissues. Deletion of each kindlin gene in fish, mice and/or humans is associated with significant functional impairments. To expand, deficiencies of K1 and K3 in humans have profound consequences giving rise to Kindler Syndrome, a skin fragility disorder, and LAD III, a disease associated with bleeding, susceptibility to infections and bone abnormalities [44,45,46]. Mice with inactivation of kindlins exhibit phenotypes that recapitulate these human diseases (K1 or K3 deficiencies). There is no reported deficiency of K2 in humans, and K2 gene inactivation is embryonically lethal in mice (day e7.5) and zebrafish [47,48]. However, the tissue-specific expression patterns and functions of the kindlins have blurred with the detection of individual kindlins in unexpected locations and the presence of more than one kindlin within the same cells [26,49,50]. The involvement of the different kindlins in many different cancers further blurred these demarcations. For example, K3 levels are elevated in non-myeloid tumors such as breast cancer [51].

Crystal structures of kindlins, including their complex with integrin β CT have been reported [52,53,54,55]. These studies have suggested that kindlins can multimerize. In the case of K2, dimerization appears to favor integrin activation [52,53] while trimerization of K3 impedes integrin activation [54]. All of these crystal structures have verified the central role of the QW motif in the F3 subdomains of the three kindlins in integrin CT binding. Biophysical studies have yielded variable results as to the significance of multimerization in integrin activation [56,57].

Several different mechanisms have been proposed as to how the binding of kindlins to beta subunits activates integrins, but consensus has yet to be reached. Ye et al. [58] used nanodiscs and concluded that talin was sufficient for integrin activation and found no effect of kindlin in this system. In studies of a β2 integrin, Lefort et al. [59] suggested that talin led to integrin extension while kindlin-3 opened up the headpiece. Ye et al. [60] suggested that kindlins’ function in integrin activation depends on their capacity to cluster integrins, i.e., the primary role of talin was to induce affinity modulation while kindlins’ primary role was to induce avidity modulation by clustering integrins. This model has received wide acceptance. Nevertheless, many of the same authors again concluded that talin induces extension of the integrin stalks and kindlin opens the headpiece leading to functional β2 integrins and implicated the PH domain of K3 as essential for this pathway of integrin activation [37]. However, earlier studies found that still other domains of kindlins are involved in integrin activation [28,61]. The distinct roles of talin and kindlin in integrin activation were supported by the study of Theodosiou et al. [62] who found that inactivation of the genes for talin or K2 both impaired integrin function but their contribution to adhesive functions could be distinguished. The distinct role of talin and kindlin in integrin activation was also supported by correlation microscopy which showed that K2 localization to nascent adhesions preceded talin recruitment [63]. The role of kindlins in the induction of conformational change in integrin activation and in integrin clustering are not necessarily incompatible, but the mechanism by which kindlins induce integrin clustering remains to be explained. The demonstration that paxillin and ADAP interact with both kindlins and talin and may link the two integrin binding partners [62,64] and allow force transmission to the cytoskeleton [65,66] also needs to be integrated into a cohesive model.

### 1.2. Phosphorylation of Kindlins

Mass spectrometry and antibodies specific for phosphorylated amino acids have been used to identify a number of phosphorylation sites in each of the three kindlins. These studies were either targeted, focused on a particular kindlin or derived from global phosphoproteomic studies in specific cell types. Multiple phosphorylation sites have been identified in each of the three kindlins; and serine, threonine and tyrosine modifications have all have been identified (Table 1). To date, of the 65 phosphorylation sites, 14 have been identified in K1, 32 in K2 and 19 in K3. These are primarily pS, 38, with 15 pT and 12 pY. The modified residues reside in all major subdomains of the kindlins (Table 1). The functional significance of most of the individual phosphorylation sites remains to be established, but this is the truth for many of the phosphorylation sites in proteins. The phosphorylation sites in the kindlins occur primarily at nonconserved residues; therefore, attempts to align the phosphorylated residues in the kindlins have not been productive. To date, none of the phosphorylation sites in three kindlins has been directly implicated in oligomerization, membrane docking or interactions with known binding partners, including the binding of the phosphorylated and non-phosphorylated forms of S^481^ of K3 to integrin β3 CT [67].

The eight phosphorylation sites in kindlins depicted in Figure 1 have been assigned specific functions, and we discuss these briefly below. In K1, a particular function has been assigned to T^8^ and T^30^ within the F0 subdomain in the regulation of mitotic spindles [68]. Orientation of mitotic spindles is known to be regulated by β1-integrins, and disruption of K1 mimics the effect of the β1-integrin depletion on spindle phenotype. K1 is exclusively phosphorylated by Plk1 at centrosomes. While K1 phosphorylation of T^8^ and T^30^ is pivotal for normal spindle formation, these PTM are not required for integrin activation as demonstrated using a phosphorylation resistant K1^T8T30/AA^ mutant. K2 has also been shown to control mitotic spindle assembly through a mechanism distinct from that of K1 and depends on α-tubulin acetylation, AKT serine/threonine kinase or paxillin [69].

Several functionally important phosphorylation sites have been identified in K2 (Figure 1). Cell adhesion-dependent K2-Src interaction and phosphorylation of K2 on Y^193^ by Src was shown to be an important event in regulating integrin outside-in signaling which controls cell migration and proliferation. Focal adhesion kinase-induced recruitment of Src to focal adhesions leads to Src activation and K2 phosphorylation and in turn regulates tyrosine phosphorylation of paxillin and cell behavior [70]. K2 phosphorylation on Y^193^ also promotes recruitment of migfilin to focal adhesion, and this event plays an important role in regulation of Src activity in focal adhesions [71]. K2 phosphorylation was also shown to be important in the regulation of invadopodia formation and protease-induced remodeling of extracellular matrix in cancer cells [72]. Phosphoproteomic analyses of breast cancer cell line MDA-MB-231 plated on invadopodia-inducing dense fibrillar collagen identified several K2 phosphorylation sites: S^159^, S^181^ and S^666^. Mutation of these serines to alanines inhibited the formation of invadopodia in MDA-231 cells on dense fibrillary collagen; conversely, phospho-mimetic mutants stimulated the formation of invadopodia even on low-density collagen [72]. In colorectal cancer, K2 is phosphorylated on S^159^ by serine/threonine kinase IkB kinase subunit epsilon (IKKe), and K2 phosphorylation-promoted invadopodia formation and metastasis of colorectal cancer [73].

Agonist-induced K3 phosphorylation on T^482^ or S^484^, which reside in a hyper-variable region of K3, was first identified in hematopoietic cells including human platelets, human erythroleukemia HEL cells and mouse macrophage-like RAW 267.4 cells [67]. Phosphorylation of K3 in hematopoietic cells affected both inside-out and outside-in integrin signaling across αIIbβ3 and α5β1 integrins, and affected specific functions of K3 [74]. Two independent studies implicated protein kinase C in phosphorylation of these residues [67,75] although there is some ambiguity as to which residue is modified. An antibody specific for pS^484^ reacted with agonist-stimulated platelets and HEL cells, whereas an antibody to pT^482^ did not [74]. However, phosphorylation of T^482^ was identified in the T lymphocyte Jurkat cell line [76]. In neutrophils, K3 phosphorylation of S^484^ was dependent on integrin-linked kinase binding to K3, and K3 phosphorylation was crucial for αLβ2-induced neutrophil adhesion and recruitment [75]. K3 phosphorylation on T^482^ and/or S^484^ is also critical for its function as a tumor promoter in breast cancer cells, where K3 and its phosphorylation contributed to its tumor promoter activity [74].

## 2. Other Post-Translational Modifications of Kindlins and Concluding Remarks

The importance of phosphorylation in controlling the functions of kindlins and their role in integrin activation is just beginning to be recognized. It remains to be seen if phosphorylation of individual residues differs in different cell types and exerts cell-specific effects. This is likely to mark the next step in this emerging area. This commentary focused on phosphorylation per se but other PTM of kindlins can also be anticipated. One such PTM, ubiquitination of K2, which has functional consequences, has already been reported [77]. Talin also has been reported to be a target of ubiquitination [78] although this result was not reproduced [77]. Other ubiquinylation sites are predicted in the kindlins (see PhosphoSitePlus^®^ (www.phosphosite.org, accessed on 22 March 2021)). The proteolysis of kindlins represents another line of PTM. Calpain-induced cleavage of K3 in leukocytes was reported [79], and, in general, kindlins appear to be sensitive to proteolysis in cells providing another level of regulation. Thus, we anticipate that the PTM of kindlins identified in this commentary is likely to be harbingers of others to come.

## Figures and Tables

**Table 1:** Phosphorylated amino acids in kindlins: Compiled from: PhosphoSitePlus^®^ (www.phosphosite.org (accessed on 22 March 2021)).

Domain	Kindlin-1	Kindlin-2	Kindlin-3
F0	T^8^, S^11^,T^30^	T^32^, T^36^, S^59^	T^6^, S^8^, Y^11^, S^14^, S^31^
F1	S^169^, S^170^, S^174^, S^179^, Y^191^	T^129^, S^159^, T^172^, S^175^, S^177^, Y^179^, S^180^, S^181^, Y^185^, S^186^, T^192^, Y^193^, S^199^, T^205^, S^258^	Y^162^, S^218^
F2	S^361^, Y^392^, Y^452^	S^339^, S^351^, Y^378^, Y^395^, S^405^, S^414^, T^416^, S^435^	S^337^, S^345^, T^361^, S^428^, T^482^, S^484^, Y^504^
F3	S^506^, S^520^, T^633^	S^523^, T^536^, S^550^, Y^590^, S^610^, S^666^	T^591^, S^595^, S^622^, S^625^, S^642^

## Data Availability

All data are available in publically available databases or upon request to E.F.P.

## References

[1] Ginsberg M.H., Du X., Plow E.F. (1992). Inside-out signalling. Curr. Opin. Cell Biol..

[2] Li Z., Delaney M.K., O’Brien K.A., Du X. (2010). Signaling During Platelet Adhesion and Activation. Arter. Thromb. Vasc. Biol..

[3] Durrant T.N., van den Bosch M.T., Hers I. (2017). Integrin alphaIIbbeta3 outside-in signaling. Blood.

[4] Ginsberg M.H. (2014). Integrin activation. BMB Rep..

[5] Humphries J.D., Chastney M.R., A Askari J., Humphries M.J. (2019). Signal transduction via integrin adhesion complexes. Curr. Opin. Cell Biol..

[6] Horton E.R., Byron A., Askari J.A., Ng D.H.J., Millon-Frémillon A., Robertson J., Koper E.J., Paul N.R., Warwood S., Knight D.P. (2015). Definition of a consensus integrin adhesome and its dynamics during adhesion complex assembly and disassembly. Nat. Cell Biol..

[7] Vinogradova O., Velyvis A., Velyviene A., Hu B., Haas T.A., Plow E.F., Qin J. (2002). A structural mechanism of integrin aIIbb3 “inside-out” activation as regulated by its cytoplasmic face. Cell.

[8] Zhu L., Plow E.F., Qin J. (2021). Initiation of focal adhesion assembly by talin and kindlin: A dynamic view. Protein Sci..

[9] Zhu L., Liu H., Lu F., Yang J., Byzova T.V., Qin J. (2019). Structural Basis of Paxillin Recruitment by Kindlin-2 in Regulating Cell Adhesion. Structure.

[10] Gao J., Huang M., Lai J., Mao K., Sun P., Cao Z., Hu Y., Zhang Y., Schulte M.L., Jin C. (2017). Kindlin supports platelet integrin alphaIIbbeta3 activation by interacting with paxillin. J. Cell Sci..

[11] Kasirer-Friede A., Kang J., Kahner B., Ye F., Ginsberg M.H., Shattil S.J. (2014). ADAP interactions with talin and kindlin promote platelet integrin alphaIIbbeta3 activation and stable fibrinogen binding. Blood.

[12] Das M., Ithychanda S.S., Qin J., Plow E.F. (2011). Migfilin and Filamin as Regulators of Integrin Activation in Endothelial Cells and Neutrophils. PLoS ONE.

[13] Bouvard D., Aszodi A., Kostka G., Block M.R., Albigès-Rizo C., Fässler R. (2007). Defective osteoblast function in ICAP-1-deficient mice. Development.

[14] Ithychanda S.S., Qin J. (2011). Evidence for Multisite Ligand Binding and Stretching of Filamin by Integrin and Migfilin. Biochemistry.

[15] Liu J., Das M., Yang J., Ithychanda S.S., Yakubenko V.P., Plow E.F., Qin J. (2015). Structural mechanism of integrin inactivation by filamin. Nat. Struct. Mol. Biol..

[16] Rantala J.K., Pouwels J., Pellinen T., Veltel S., Laasola P., Mattila E., Potter C.S., Duffy T., Sundberg J.P., Kallioniemi O. (2011). SHARPIN is an endogenous inhibitor of beta1-integrin activation. Nat. Cell Biol..

[17] Gao J., Bao Y., Ge S., Sun P., Sun J., Liu J., Chen F., Han L., Cao Z., Qin J. (2019). Sharpin suppresses beta1-integrin activation by complexing with the beta1 tail and kindlin-1. Cell Commun. Signal..

[18] Han J., Rose D.M., Woodside D.G., Goldfinger L.E., Ginsberg M.H. (2003). Integrin alpha 4 beta 1-dependent T cell migration requires both phosphorylation and dephosphorylation of the alpha 4 cytoplasmic domain to regulate the reversible binding of paxillin. J. Biol. Chem..

[19] Jenkins A.L., Nannizzi-Alaimo L., Silver D., Sellers J.R., Ginsberg M.H., Law D.A., Phillips D.R. (1998). Tyrosine phosphorylation of the b3 cytoplasmic domain mediates integrin-cytoskeletal interactions. J. Biol. Chem..

[20] Gahmberg C.G., Gronholm M., Uotila L.M. (2014). Regulation of integrin activity by phosphorylation. Adv. Exp. Med. Biol..

[21] Phillips D.R., Nannizzi-Alaimo L., Prasad K.S. (2001). Beta3 tyrosine phosphorylation in alphaIIbbeta3 (platelet membrane GP IIb-IIIa) outside-in integrin signaling. Thromb. Haemost..

[22] Mitra S.K., Schlaepfer D.D. (2006). Integrin-regulated FAK-Src signaling in normal and cancer cells. Curr. Opin. Cell Biol..

[23] Turner E.C., Pavalko F.M., Burridge K. (1989). The role of phosphorylation and limited proteolytic cleavage of talin and vinculin in the disruption of focal adhesion integrity. J. Biol. Chem..

[24] Ratnikov B., Ptak C., Han J., Shabanowitz J., Hunt D.F., Ginsberg M.H. (2005). Talin phosphorylation sites mapped by mass spectrometry. J. Cell Sci..

[25] Larjava H., Plow E.F., Wu C. (2008). Kindlins: Essential regulators of integrin signalling and cell–matrix adhesion. EMBO Rep..

[26] Rognoni E., Ruppert R., Fässler R. (2016). The kindlin family: Functions, signaling properties and implications for human disease. J. Cell Sci..

[27] Malinin N.L., Plow E.F., Byzova T.V. (2010). Kindlins in FERM adhesion. Blood.

[28] Hirbawi J., Bialkowska K., Bledzka K.M., Liu J., Fukuda K., Qin J., Plow E.F. (2017). The extreme C-terminal region of kindlin-2 is critical to its regulation of integrin activation. J. Biol. Chem..

[29] Shi X., Ma Y.-Q., Tu Y., Chen K., Wu S., Fukuda K., Qin J., Plow E.F., Wu C. (2007). The MIG-2/Integrin Interaction Strengthens Cell-Matrix Adhesion and Modulates Cell Motility. J. Biol. Chem..

[30] Rensen S.S.M., Doevendans P.A.F.M., Van Eys G.J.J.M. (2007). Regulation and characteristics of vascular smooth muscle cell phenotypic diversity. Neth. Hearth J..

[31] Qu H., Tu Y., Shi X., Larjava H., Saleem M.A., Shattil S.J., Fukuda K., Qin J., Kretzler M., Wu C. (2011). Kindlin-2 regulates podocyte adhesion and fibronectin matrix deposition through interactions with phosphoinositides and integrins. J. Cell Sci..

[32] Perera D., Ma Y.-Q., Yang J., Hirbawi J., Plow E.F., Qin J. (2011). Membrane Binding of the N-Terminal Ubiquitin-Like Domain of Kindlin-2 Is Crucial for Its Regulation of Integrin Activation. Structure.

[33] Liu J., Fukuda K., Xu Z., Ma Y.-Q., Hirbawi J., Mao X., Wu C., Plow E.F., Qin J. (2011). Structural Basis of Phosphoinositide Binding to Kindlin-2 Protein Pleckstrin Homology Domain in Regulating Integrin Activation. J. Biol. Chem..

[34] Liu Y., Zhu Y., Ye S., Zhang R. (2012). Crystal structure of kindlin-2 PH domain reveals a conformational transition for its membrane anchoring and regulation of integrin activation. Protein Cell.

[35] Metcalf D.G., Moore D.T., Wu Y., Kielec J.M., Molnar K., Valentine K.G., Wand A.J., Bennett J.S., DeGrado W.F. (2010). NMR analysis of the alphaIIb beta3 cytoplasmic interaction suggests a mechanism for integrin regulation. Proc. Natl. Acad. Sci. USA.

[36] Hart R., Stanley P., Chakravarty P., Hogg N. (2013). The Kindlin 3 Pleckstrin Homology Domain Has an Essential Role in Lymphocyte Function-associated Antigen 1 (LFA-1) Integrin-mediated B Cell Adhesion and Migration. J. Biol. Chem..

[37] Wen L., Marki A., Roy P., McArdle S., Sun H., Fan Z., Gingras A.R., Ginsberg M.H., Ley K. (2021). Kindlin-3 recruitment to the plasma membrane precedes high affinity beta2 integrin and neutrophil arrest from rolling. Blood.

[38] Bledzka K., Liu J., Xu Z., Perera H.D., Yadav S.P., Bialkowska K., Qin J., Ma Y., Plow E.F. (2012). Spatial coordination of kindlin-2 with talin head domain in interaction with integrin beta cytoplasmic tails. J. Biol. Chem..

[39] Plow E.F., Das M., Bialkowska K., Sossey-Alaoui K. (2016). Of Kindlins and Cancer. Discoveries.

[40] Plow E.F., Qin J. (2019). The Kindlin Family of Adapter Proteins. Circ. Res..

[41] Ussar S., Wang H.-V., Linder S., Fässler R., Moser M. (2006). The Kindlins: Subcellular localization and expression during murine development. Exp. Cell Res..

[42] Meves A., Stremmel C., Gottschalk K., Fässler R. (2009). The Kindlin protein family: New members to the club of focal adhesion proteins. Trends Cell Biol..

[43] Karaköse E., Schiller H.B., Fässler R. (2010). The kindlins at a glance. J. Cell Sci..

[44] Ussar S., Moser M., Widmaier M., Rognoni E., Harrer C., Genzel-Boroviczény O., Fässler R. (2008). Loss of Kindlin-1 Causes Skin Atrophy and Lethal Neonatal Intestinal Epithelial Dysfunction. PLoS Genet..

[45] Moser M., Nieswandt B., Ussar S., Pozgajova M., Fässler R. (2008). Kindlin-3 is essential for integrin activation and platelet aggregation. Nat. Med..

[46] Malinin N.L., Pluskota E., Byzova T.V. (2012). Integrin signaling in vascular function. Curr. Opin. Hematol..

[47] Montanez E., Ussar S., Schifferer M., Bösl M., Zent R., Moser M., Fässler R. (2008). Kindlin-2 controls bidirectional signaling of integrins. Genes Dev..

[48] Dowling J.J., Gibbs E., Russell M., Goldman D., Minarcik J., Golden J.A., Feldman E.L. (2008). Kindlin-2 Is an Essential Component of Intercalated Discs and Is Required for Vertebrate Cardiac Structure and Function. Circ. Res..

[49] Bialkowska K., Ma Y.-Q., Bledzka K., Sossey-Alaoui K., Izem L., Zhang X., Malinin N., Qin J., Byzova T., Plow E.F. (2010). The Integrin Co-activator Kindlin-3 Is Expressed and Functional in a Non-hematopoietic Cell, the Endothelial Cell. J. Biol. Chem..

[50] Harburger D.S., Bouaouina M., Calderwood D.A. (2009). Kindlin-1 and -2 directly bind the C-terminal region of beta integrin cytoplasmic tails and exert integrin-specific activation effects. J. Biol. Chem.

[51] Sossey-Alaoui K., Pluskota E., Davuluri G., Bialkowska K., Das M., Szpak D., Lindner D.J., Downs-Kelly E., Thompson C.L., Plow E.F. (2014). Kindlin-3 enhances breast cancer progression and metastasis by activating Twist-mediated angiogenesis. FASEB J..

[52] Li H., Deng Y., Sun K., Yang H., Liu J., Wang M., Zhang Z., Lin J., Wu C., Wei Z. (2017). Structural basis of kindlin-mediated integrin recognition and activation. Proc. Natl. Acad. Sci. USA.

[53] Sun J., Xiao D., Ni Y., Zhang T., Cao Z., Xu Z., Nguyen H., Zhang J., White G.C., Ding J. (2020). Structure basis of the FERM domain of kindlin-3 in supporting integrin alphaIIbbeta3 activation in platelets. Blood Adv..

[54] Bu W., Levitskaya Z., Loh Z.Y., Jin S., Basu S., Ero R., Yan X., Wang M., Ngan S.F.C., Sze S.K. (2020). Structural basis of human full-length kindlin-3 homotrimer in an auto-inhibited state. PLoS Biol..

[55] Yates L.A., Lumb C.N., Brahme N.N., Zalyte R., Bird L.E., De Colibus L., Owens R.J., Calderwood D.A., Sansom M.S.P., Gilbert R.J.C. (2012). Structural and Functional Characterization of the Kindlin-1 Pleckstrin Homology Domain. J. Biol. Chem..

[56] Yates L.A., Füzéry A.K., Bonet R., Campbell I.D., Gilbert R.J.C. (2012). Biophysical analysis of Kindlin-3 reveals an elongated conformation and maps integrin binding to the membrane-distal beta-subunit NPXY motif. J. Biol. Chem..

[57] Kadry Y.A., Maisuria E.M., Huet-Calderwood C., Calderwood D.A. (2020). Differences in self-association between kindlin-2 and kindlin-3 are associated with differential integrin binding. J. Biol. Chem..

[58] Ye F., Hu G., Taylor D., Ratnikov B., Bobkov A.A., McLean M.A., Sligar S.G., Taylor K.A., Ginsberg M.H. (2010). Recreation of the terminal events in physiological integrin activation. J. Cell Biol..

[59] Lefort C.T., Rossaint J., Moser M., Petrich B.G., Zarbock A., Monkley S.J., Critchley D.R., Ginsberg M.H., Fässler R., Ley K. (2012). Distinct roles for talin-1 and kindlin-3 in LFA-1 extension and affinity regulation. Blood.

[60] Ye F., Petrich B.G., Anekal P., Lefort C.T., Kasirer-Friede A., Shattil S.J., Moser M., Fässler R., Ginsberg M.H. (2013). The mechanism of kindlin-mediated activation of integrin alphaIIbbeta3. Curr. Biol..

[61] Xu Z., Gao J., Hong J., Ma Y.-Q. (2013). Integrity of kindlin-2 FERM subdomains is required for supporting integrin activation. Biochem. Biophys. Res. Commun..

[62] Theodosiou M., Widmaier M., Böttcher R.T., Rognoni E., Veelders M., Bharadwaj M., Lambacher A., Austen K., Müller D.J., Zent R. (2016). Kindlin-2 cooperates with talin to activate integrins and induces cell spreading by directly binding paxillin. eLife.

[63] Bachir A.I., Zareno J., Moissoglu K., Plow E.F., Gratton E., Horwitz A.R. (2014). Integrin-Associated Complexes Form Hierarchically with Variable Stoichiometry in Nascent Adhesions. Curr. Biol..

[64] Kasirer-Friede A., Moran B., Nagrampa-Orje J., Swanson K., Ruggeri Z.M., Schraven B., Neel B.G., Koretzky G., Shattil S.J. (2007). ADAP is required for normal alphaIIbbeta3 activation by VWF/GP Ib-IX-V and other agonists. Blood.

[65] Nordenfelt P., Elliott H.L., Springer T.A. (2016). Coordinated integrin activation by actin-dependent force during T-cell migration. Nat. Commun..

[66] Goult B.T., Yan J., Schwartz M.A. (2018). Talin as a mechanosensitive signaling hub. J. Cell Biol..

[67] Bialkowska K., Byzova T.V., Plow E.F. (2015). Site-specific phosphorylation of kindlin-3 protein regulates its capacity to control cellular responses mediated by integrin alphaIIbbeta3. J. Biol. Chem..

[68] Patel H., Zich J., Serrels B., Rickman C., Hardwick K.G., Frame M.C., Brunton V.G. (2013). Kindlin-1 regulates mitotic spindle formation by interacting with integrins and Plk-1. Nat. Commun..

[69] Tan H.-F., Tan S.-M. (2020). The focal adhesion protein kindlin-2 controls mitotic spindle assembly by inhibiting histone deacetylase 6 and maintaining α-tubulin acetylation. J. Biol. Chem..

[70] Qu H., Tu Y., Guan J.-L., Xiao G., Wu C. (2014). Kindlin-2 Tyrosine Phosphorylation and Interaction with Src Serve as a Regulatable Switch in the Integrin Outside-in Signaling Circuit. J. Biol. Chem..

[71] Liu Z., Lu D., Wang X., Wan J., Liu C., Zhang H. (2015). Kindlin-2 phosphorylation by Src at Y193 enhances Src activity and is involved in Migfilin recruitment to the focal adhesions. FEBS Lett..

[72] Artym V.V., Swatkoski S., Matsumoto K., Campbell C.B., Petrie R.J., Dimitriadis E.K., Li X., Mueller S.C., Bugge T.H., Gucek M. (2015). Dense fibrillar collagen is a potent inducer of invadopodia via a specific signaling network. J. Cell Biol..

[73] Liu G., Bao Y., Liu C., Zhu Q., Zhao L., Lu X., Zhu Q., Lv Y., Bai F., Wen H. (2020). IKKepsilon phosphorylates kindlin-2 to induce invadopodia formation and promote colorectal cancer metastasis. Theranostics.

[74] Bialkowska K., Sossey-Alaoui K., Pluskota E., Izem L., Qin J., Plow E.F. (2019). Site-specific phosphorylation regulates the functions of kindlin-3 in a variety of cells. Life Sci. Alliance.

[75] Margraf A., Germena G., Drexler H.C.A., Rossaint J., Ludwing N., Prystaj B., Mersamann S., Thomas K., Block H., Gottschilch W. (2020). The integrin linked kinase is required for chemokine-triggered high affinity conformation of neutrophil beta2-integrin LFA1. Blood.

[76] Ruperez P., Gago-Martinez A., Burlingame A.L., Oses-Prieto J.A. (2012). Quantitative Phosphoproteomic Analysis Reveals a Role for Serine and Threonine Kinases in the Cytoskeletal Reorganization in Early T Cell Receptor Activation in Human Primary T Cells. Mol. Cell. Proteom..

[77] Wei X., Wang X., Zhan J., Chen Y., Fang W., Zhang L., Zhang H. (2017). Smurf1 inhibits integrin activation by controlling Kindlin-2 ubiquitination and degradation. J. Cell Biol..

[78] Huang C., Rajfur Z., Yousefi N., Chen Z., Jacobson K., Ginsberg M.H. (2009). Talin phosphorylation by Cdk5 regulates Smurf1-mediated talin head ubiquitylation and cell migration. Nat. Cell Biol..

[79] Zhao Y., Malinin N.L., Meller J., Ma Y., West X.Z., Bledzka K., Qin J., Podrez E.A., Byzova T.V. (2012). Regulation of Cell Adhesion and Migration by Kindlin-3 Cleavage by Calpain. J. Biol. Chem..

